# The Expression of Bcl-2 and BID in Gastric Cancer Cells

**DOI:** 10.1155/2014/953203

**Published:** 2014-02-19

**Authors:** Mariusz Gryko, Anna Pryczynicz, Konrad Zareba, Bogusław Kędra, Andrzej Kemona, Katarzyna Guzińska-Ustymowicz

**Affiliations:** ^1^Second Department of General and Gastroenterological Surgery, Medical University of Bialystok, M. Sklodowskiej-Curie 24a Street, 15-276 Bialystok, Poland; ^2^Department of General Pathomorphology, Medical University of Bialystok, M. Sklodowskiej-Curie 24a Street, 15-276 Bialystok, Poland

## Abstract

*Background.* Bcl-2 and BID play a major role in the process of apoptosis and their dysfunction underlies carcinogenesis. The study objective was to assess the expression of Bcl-2 and BID in gastric cancer cells in correlation with chosen clinicopathological parameters, presence of *Helicobacter pylori* infection, and patients' survival. *Materials and Methods.* The study involved 88 patients operated on for gastric cancer. The expressions of Bcl-2 and BID were determined immunohistochemically. *Results.* Positive Bcl-2 expression was found in 55.7% and, BID in 53.6% of patients. The Bcl-2 expression correlated with stage pT3 and T4 gastric cancer (*P* < 0.05), with the intestinal type according to Lauren (*P* < 0.001), ulcerated type according to Bormann's classification (*P* < 0.01), and with local lymph node metastases (*P* < 0.05). *Conclusion.* The Bcl-2 protein plays a key role in the process of gastric cancer formation and is associated with the growth of definite types of gastric cancer.

## 1. Introduction

Apoptosis is a physiological programmed cell death that plays a major role in the process of carcinogenesis. It involves a number of proteins, including Bcl-2 and BID, which belong to the Bcl-2 family but have different functions. The Bcl-2 protein inhibits the mitochondrial pathway of apoptosis, interacting with other members of the Bcl-2 family. Its increased expression shifts the balance between the pro- and antiapoptotic factors toward cell survival. The BID protein is a member of another group of the Bcl-2 family. It activates apoptosis and at the same time integrates two main apoptotic routes, connecting the membranous (external) and mitochondrial (internal) pathways. However, its role in the pathogenesis of cancer is still poorly elucidated. Apoptotic disorders are associated with the development of many cancers, including that of the stomach [1–5]. *Helicobacter pylori* infection seems to play a major role in this process [6–9].

The study objective was to assess the expressions of Bcl-2 and BID in gastric cancer cells depending on the sex and age of patients, histological type of tumor according to Lauren, macroscopic type according to Bormann, tumor grade (G), advancement stage (pT), tumor location in the stomach, the presence of *Helicobacter pylori* infection, local lymph node involvement, and the effect of Bcl-2 and BID expression in cancer cells on postoperative survival. Additionally, correlations were assessed between Bcl-2 and BID for their role in the process of apoptosis.

## 2. Materials and Methods

### 2.1. Patients

The study involved 88 patients operated on for gastric cancer in the Second Department of General and Gastroenterological Surgery, Medical University of Bialystok, in the years 2000–2006 (Table 1). The patients had received neither radiotherapy nor chemotherapy prior to surgery.

The immunohistochemical investigations were performed using archival material consisting of paraffin blocks with the presence of gastric cancer tissues. Tumor stage and clinicomorphological features were assessed based on the postoperative examination of the surgically resected specimens (stomach with lymph nodes) and the intraoperative picture according to the AJCC (American Joint Committee on Cancer) criteria [10].

The study was conducted as part of a statutory project approved by the Bioethics Committee, Medical University of Bialystok.

### 2.2. Immunohistochemical Analysis

The expression levels of Bcl-2 and BID were determined by immunohistochemistry in gastric cancer cells. Formalin-fixed and paraffin-embedded cancer tissue specimens were cut with a microtome into 4 *μ*m sections, which were then deparaffinized in xylenes and hydrated at decreasing alcohol concentrations.

For both proteins, antigens were retrieved by heating in citrate buffer (pH = 6.0) for 15 min. After rinsing in PBS buffer (pH = 7.4) the sections were incubated with primary antibodies (Bcl-2-goat polyclonal antibody, clone N-19, Sc-492-G, Santa Cruz Biotechnology and BID-goat polyclonal antibody, clone N-19, Sc-6539, Santa Cruz Biotechnology) at 1 : 100 dilution for 1 hour at room temperature. The polymer En Vision FLEX Visualization System kit (DAKO, Poland) was used as a detection system. The antigen-antibody complex was visualized using DAB chromogen (S3000, DAKO, Poland).

Positive reaction of proteins was observed in cytoplasm of cancer cells. The expression of Bcl-2 in cancer cells was assessed in a semiquantitative way: it was defined positive when present in over 10% of cancer cells, whereas negative when absent or present in fewer than 10% of cancer cells [11]. Similarly, for BID the reaction was considered positive when present in over 20% of cancer cells, negative when absent or present in fewer than 20% of cancer cells [12].

### 2.3. *Helicobacter pylori*

*H. pylori* bacilli were confirmed in the antral gastric mucosa samples when stained by the modified Giemsa method.

### 2.4. Statistical Analysis

The correlation between qualitative variables was assessed by means of the exact Fisher test. In the case of more than two variants of a variable the Fisher-Freeman-Halton test was applied. Distributions of the ordinal variables were compared between two groups by means of the Mann-Whitney test and *χ* ^2^ test for trend. The correlations between two ordinal variables were described using the Kaplan-Meier curve and survivals were compared by Gehan-Wilcoxon test. In all the tests, the level of significance was considered at *P* < 0.05, with highly significant differences at *P* < 0.01. Calculations were performed using the IBM (R) SPSS Statistics 20.0 program. The program implementing an adequate algorithm was employed for the Fisher-Freeman-Halton test [13].

## 3. Results

**Figure 1:**
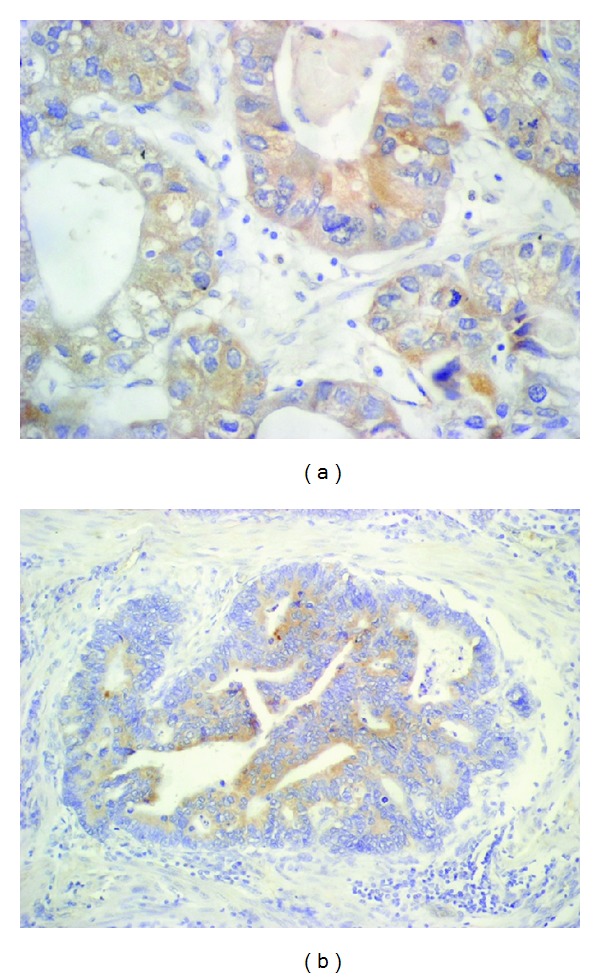
(a) Positive expression of Bcl-2 in cytoplasm of gastric cancer cells (IHC stain, ×20). (b) Positive expression of BID in cytoplasm of gastric cancer cells (IHC stain, ×40).

Positive Bcl-2 expression was found in 49/88 patients (55.7%), whereas BID in 45/84 (53.6%) (Figure 1, Table 1).

Positive Bcl-2 expression was significantly more frequent in patients with more advanced gastric cancer (T3, T4) than in those with less advanced tumors (T1, T2), (37/58; 63.8% versus 12/30; 40%, *P* < 0.05). No such correlation was noted for BID.

The Bcl-2 protein was more frequently expressed in cancer cells in patients with the intestinal type of gastric cancer than in the diffuse type, the difference being statistically significant (42/59; 71.2% versus 7/21; 25.0%, *P* < 0.001). The expression of BID was similar in both histological types.

The expression of Bcl-2 was most frequent in patients with the ulcerated type with sharply demarcated margins according to Bormann (type II), completely missing in patients with the polyp type (type I), and moderate in those with ulcerated infiltrative type (type III) and “linitis plastica” (type IV) (15/21; 71.4% versus 0/7; 0.00% versus 26/45; 57.8% versus 8/15; 53.3%, *P* < 0.05). In the case of BID, the differences between the respective forms of cancer according to Bormann were not statistically significant.

Higher expression of Bcl-2 was noted in cancer cells in patients with local lymph node involvement as compared to the metastasis-free patients (19/26; 73.1% versus 30/62; 48.4%, *P* < 0.05). No such correlation was found for BID.

No statistically significant differences were observed in the expressions of Bcl-2 and BID depending on age, sex, grade (G), location in the stomach, and *Helicobacter pylori* infection (Table 1).

Postoperative survival of patients was assessed based on the Kaplan-Meier curve (patients with positive and negative expressions of Bcl-2 and BID) and then compared using the Gehan-Wilcoxon test. The expression levels of the proteins studied had no effect on the patients' survival time.

The correlations between the expression levels of Bcl-2 and BID in gastric cancer cells were found to be statistically insignificant.

## 4. Discussion

Apoptotic disorders underlie carcinogenesis. *Helicobacter pylori* infection and the associated protein dysfunctions, including antiapoptotic Bcl-2 and proapoptotic BID, have a major role in gastric cancer [1–9]. The assessment of their expression in gastric cancer cells in relation to morphological and histological factors as well as *H. pylori *infection may help elucidate the formation and growth of various forms of gastric cancer.

We found positive expression of Bcl-2 in 55.7% and BID in 53.6% of patients with gastric cancer. Results reported by other authors show a large divergence. Tsamandas et al. [14] noted a positive expression of Bcl-2 in as many as 67% of patients. In turn, Saegusa et al. [15] found Bcl-2 expression only in 14%, Tsamandas et al. [14] in 22.2%, Yildirim et al. [16] in 23.8%, Smith et al. [17] in 23%, and van der Woude et al. [18] found no Bcl-2 (0%) in gastric cancer patients. Positive expression of BID was reported by Smith et al. in 66% of patients [17].

We observed significantly higher frequency of antiapoptotic Bcl-2 expression in patients with more advanced tumors as compared to an earlier stage (63.8% versus 40.0%, *P* < 0.05). However, the literature data are also discrepant. Some authors described more frequent expression of this protein in early forms of cancer [14, 15]. Others found no differences in Bcl-2 expression depending on tumor stage [19–21]. However, Liu et al. noted more frequent expression of the protein in stage II according to UICC as compared to stages I and III [22]. Our findings can be explained by increased survival capacity acquired by cancer cells with high expression of antiapoptotic Bcl-2 or by possible changes in the protein profile in the course of cancer growth.

Referring to Lauren's classification, we noted a distinct difference in the frequency of Bcl-2 expression depending on tumor differentiation. It was significantly higher in the intestinal type (Lauren I), as compared to the diffuse type (Lauren II) (71.2% versus 25.0%, *P* < 0.001). Other authors described a similar tendency [12, 13, 18, 19]. Some researchers failed to confirm such a correlation [15, 18, 22]. The results might be explained by differences in the process of carcinogenesis between the respective forms of cancer in its early stages. Lack of significant differences in the expression of BID in histopathologically diverse forms of cancer (60.0% versus 42.9%, NS) can be partly explained by the observations of Lee et al. [1], who showed that BID gene mutation is rare (6%) but is associated with patients' resistance to chemotherapy.

We found considerable differences in Bcl-2 expression in patients with various macroscopic forms of cancers according to Bormann. No expression was observed (0%) in patients with the polyp type (type I), whereas the expression was the most frequent in the ulcerated type (type II) (71.4%) and moderate in infiltrating cancers (types III and IV) (57.8% and 53.3%). Reports of other authors are not explicit. Some researchers failed to show any correlations between Bcl-2 expression and the macroscopic pattern of gastric cancer [22]. Others found differences even between adenocarcinomas and various histological types of early gastric cancer, showing more frequent expression of Bcl-2 in adenomas and convex form of early cancer, as compared to the recessed form [14]. Our study confirmed the role of Bcl-2 in the formation of various forms of gastric cancer.

Unlike other authors who failed to find differences in Bcl-2 expression in cancer cells in relation to lymph node metastases [21, 22], we revealed higher frequency of Bcl-2 expression in patients with lymph node involvement. Pan et al. [23] showed a similar correlation in a group of patients with early gastric cancer, whereas Müller et al. [20] observed more frequent expression of Bcl-2 in patients without lymph node involvement. Facilitated metastasizing can be theoretically explained by greater viability of cells with high Bcl-2 expression, due to higher resistance to apoptosis. However, this should be further confirmed.

The expression of the two proteins studied had no effect on postoperative survival. Some authors presented similar results [20, 21, 24–26]; others associated more frequent expression of Bcl-2 in cancer cells with better prognosis and longer survival [16, 22, 27]. In turn, Kopp et al. showed shorter survival of patients with T1-T2 tumors with positive Bcl-2 expression [28].

*Helicobacter pylori* infection is a factor known to cause apoptotic disorders that lead to the development of gastric cancer. Most authors reported activation of apoptosis in cancer cells under the effect of *Helicobacter pylori*, with activation of its mitochondrial pathway and increased expression of BID and Bax, or antiapoptotic Bcl-2 [6, 8, 9]. We failed to confirm the differences in the expression of the proteins studied between patients with and without* Helicobacter pylori* infection in the stomach.

Concluding, the differences in the expression of the proteins in gastric cancer cells, especially Bcl-2, may indicate that they have an effect on the type of apoptotic disorders, and thus on the formation of various types of cancers. This refers both to the macroscopic and histopathologic patterns. Even though more frequent expression of this protein is associated with more differentiated forms of cancer, it does not affect patients' survival and in our opinion should not be taken into account as a prognostic factor. Increased frequency of Bcl-2 expression in more advanced tumors and in those with lymph node involvement can be explained by greater survival capacity acquired by cells with high Bcl-2 expression due to apoptosis inhibition. This may also suggest that both the formation and growth of tumor is a dynamic process, during which cancer cells undergo constant changes in the synthesis and function of many proteins, including Bcl-2. The presented results demonstrate that the formation and growth of cancer is a complex process that requires further research.

## Figures and Tables

**Table 1:** Bcl-2 and BID expression in gastric cancer cells depending on clinicopathological factors and *Helicobacter pylori* infection.

Variable	Bcl-2		*P*	BID		*P*
	(+)	(−)		(+)	(−)	
	*n* (%)			*n* (%)		
Protein expression	49 (55.7%)	39 (44.3%)		45 (53.6%)	39 (46.4%)	
Age						
<60	16 (48.5%)	17 (51.5%)	NS	16 (48.5%)	17 (51.5%)	NS
≥60	33 (60.0%)	22 (40.0%)		29 (56.9%)	22 (43.1%)	
Sex						
Female	16 (55.2%)	13 (44.8%)	NS	12 (44.4%)	15 (55.6%)	NS
Male	33 (55.9%)	26 (44.1%)		33 (57.9%)	24 (42.1%)	
Depth of infiltration (pT)						
pT1, T2	12 (40.0%)	18 (60.0%)	<0,05	11 (39.3%)	17 (60.7%)	NS
pT3, T4	37 (63.8%)	21 (36.2%)		34 (60.7%)	22 (39.3%)	
Lauren's classification						
Intestinal type	42 (71.2%)	17 (37.8%)	<0,001	33 (60.0%)	22 (40.0%)	NS
Diffuse type	7 (25.0%)	21 (75.0%)		12 (42.9%)	16 (57.1%)	
Malignancy grade (G)						
2	28 (62.2%)	17 (37.8%)	NS	27 (62.8%)	16 (37.2%)	NS
3	21 (48.8%)	22 (51.2%)		18 (43.9%)	23 (56.1%)	
Cancer location in the stomach						
Upper 1/3	2 (33.3%)	4 (66.7%)	NS	3 (50.0%)	3 (50.0%)	NS
Middle 1/3	21 (55.3%)	17 (44.7%)		18 (50.0%)	18 (50.0%)	
Lower 1/3	26 (59.1%)	18 (40.9%)		24 (57.1%)	18 (42.9%)	
Bormann's classification						
1	0 (00.0%)	7 (100%)	<0,01	4 (57.1%)	3 (42.9%)	NS
2	15 (71.4%)	6 (28.6%)		6 (33.3%)	12 (66.7%)	
3	26 (57.8%)	19 (42.2%)		28 (65.1%)	15 (34.9%)	
4	8 (53.3%)	7 (46.7%)		7 (43.8%)	9 (56.3%)	
Lymph node involvement						
Present	19 (73.1%)	7 (26.9%)	<0,05	13 (52.0%)	12 (48.0%)	NS
Absent	30 (48.4%)	32 (51.6%)		32 (54.2%)	27 (45.8%)	
*H. pylori* infection						
Present	21 (51.2%)	20 (48.8%)	NS	20 (48.8%)	21 (51.2%)	NS
Absent	23 (62.2%)	14 (37.8%)		21 (61.8%)	13 (38.2%)	

NS: nonsignificant.
